# Overtube-Assisted Foreign Body Removal: A Review of Endoscopic Management and Case Illustration

**DOI:** 10.7759/cureus.1730

**Published:** 2017-09-29

**Authors:** Andrew Ofosu, Daryl Ramai, Madhavi Reddy

**Affiliations:** 1 Department of Gastroenterology, The Brooklyn Hospital Center, Affiliate of the Mount Sinai Hospital

**Keywords:** foreign body, endoscopy, overtube, treatment management

## Abstract

The ingestion of foreign bodies is a common medical emergency seen in both adults and children. In children, the most commonly ingested foreign bodies include coins, toys, magnets, and batteries. In adults, food bolus impaction represents the most common cause of foreign body ingestion. The majority of foreign bodies pass spontaneously. Sharp or pointed objects increase the risk of perforation. Emergent endoscopic intervention is indicated in cases of esophageal obstruction, ingestion of disk batteries, and sharp pointed objects in the esophagus. Flexible endoscopy is the therapeutic method of choice for removing foreign bodies. It is preferred due to its high success rate and low risk for complications. Additionally, the use of an overtube provides gastric and esophageal protection from mucosal laceration. We present a 27-year-old male who ingested six razor blades and a curtain hook and review endoscopic management.

## Introduction

In 2012, foreign body ingestion accounted for 588,322 of emergency department visits in all age groups [1]. Most adult cases are associated with bone or meat bolus impaction. The ingestion of nonfood objects is more commonly seen in the pediatric population as well as cases associated with psychiatric illness and/or drug abuse [2]. While most foreign bodies pass spontaneously, approximately 10-20% of cases require endoscopic intervention, with few cases requiring surgery [3]. The use of an overtube is an effective and safe method for withdrawing foreign bodies among other clinical indicators. Herein, we present a 27-year-old male who ingested several foreign bodies, which were successfully and safely endoscopically removed using an overtube.

## Case presentation

**Figure 1:**
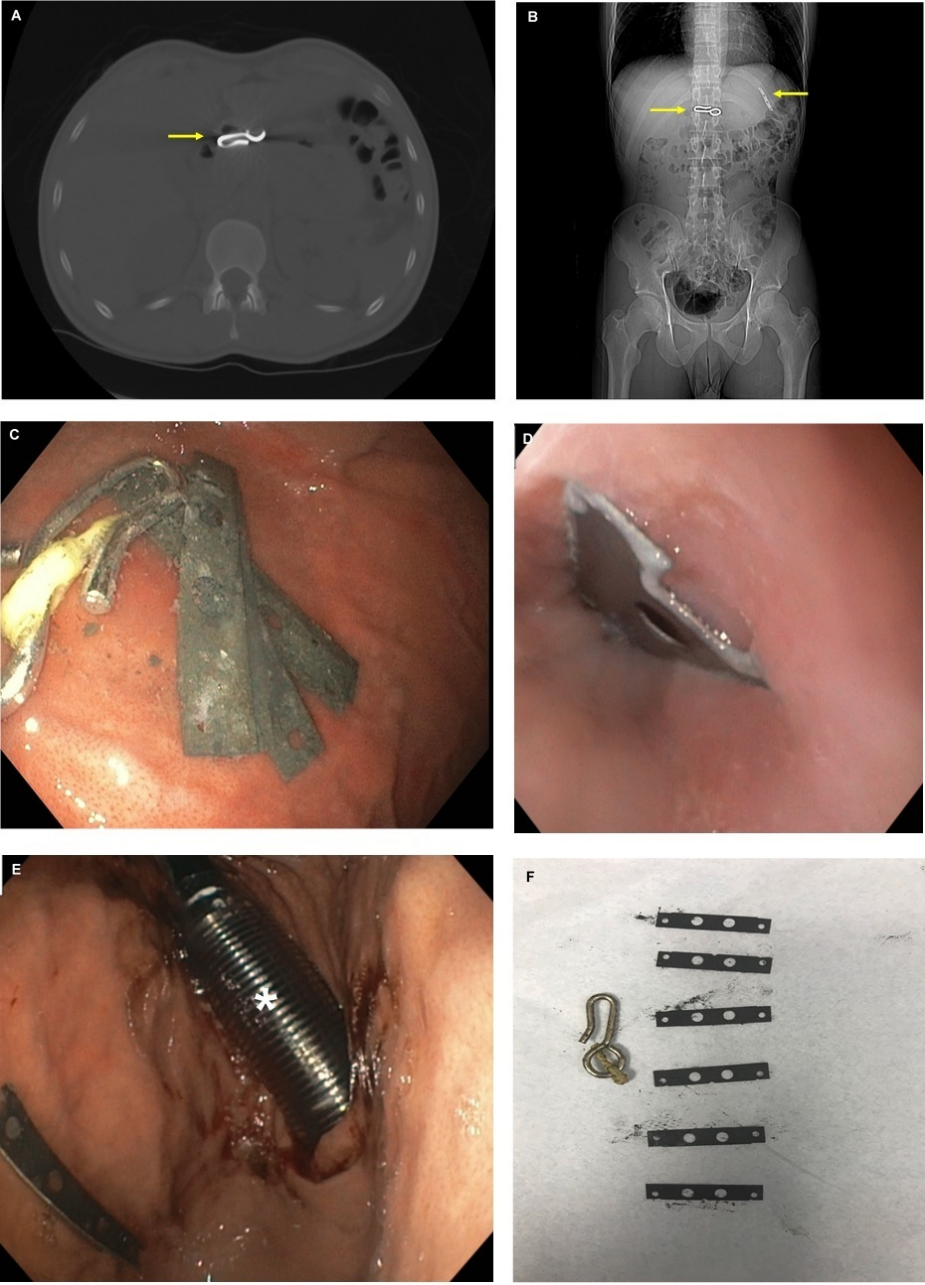
A – Coronal computed tomography scan of a curtain hook (arrow). B – Axial computed tomography scan showing a curtain hook and a single razor blade (arrows). C – Endoscopic visualization of razor blades and a curtain hook in the stomach. D – Razor blade seen at the gastroesophageal junction. E – Overtube (asterisk) was used to protect the mucosal wall during foreign body removal. F – Six razor blades and a curtain hook were sequentially removed from the body of the stomach.

A 27-year-old male with a history of psychosis presented to the emergency room with mild, sharp, intermittent lower abdominal pain. His past medical history was notable for auditory hallucinations, leading to prior suicidal attempts. The patient admitted to voices that instructed him to ingest razor blades and a metal object two days ago. Physical examination was unremarkable except for mild mid-abdominal tenderness to deep palpation without rebound tenderness or guarding. Abdominal and pelvic computed tomography (CT) scans showed a curtain hook in the gastric antrum and a metallic density razor blade in the proximal gastric body (Figure 1A, 1B). The patient had an esophagogastroduodenoscopy, which identified six razor blades (each 5 cm in length), including a razor at the gastroesophageal junction (Figure 1C, 1D). The foreign bodies were sequentially removed using an overtube and a raptor grasping device (Figure 1E, 1F). 

## Discussion

Endoscopy is the most reliable diagnostic method in cases of suspected foreign body ingestion. Since most stomach containing foreign bodies pass within 4-6 days, conservative management is preferred in asymptomatic cases. The timing of endoscopy varies depending on age, location, size, and shape of the foreign body. According to the American Society of Gastrointestinal Endoscopy (ASGE), foreign body management is divided into three categories: emergent, urgent, and nonurgent endoscopic removal [4]. Emergent cases include esophageal obstruction, disk battery in the esophagus, and sharp pointed objects in the esophagus; urgent cases include esophageal objects that are not sharp and pointed, esophageal food impaction without complete obstruction, objects > 6 cm at or above the duodenum, and magnets within endoscopic reach; and nonurgent cases include coins, objects > 2.5 cm in diameter, disk batteries in the stomach that can be observed up to 48 hours if asymptomatic (if longer than 48 hours, these batteries should be removed) [5].

Several instruments are available to the endoscopist. Flexible endoscopes are the diagnostic as well as the therapeutic method of choice used for identifying and removing foreign bodies with a success rate of > 95% and a complication rate of 0%-5% [6]. Flexible endoscopes are preferred to rigid endoscopes due to their lower risk of complications such as perforation. Additionally, double balloon enteroscopy has been successful in extracting entrapped capsules from the small intestines [7]. The choice of retrieval device is dependent on the preference of the endoscopists and includes polypectomy snares, grasping forceps, magnetic probes, retrieval baskets, transparent cap-fitting devices, which are frequently used in endoscopic mucosal resection, and overtubes.

Witzel et al. (1974) published the first case of an overtube being used to assist in removing ingested razor blades [8]. Since then, many overtube adaptations have been made. The overtube resembles a sleeve-like device, which possesses a diameter that is larger than an endoscope to enable its passage into the esophagus and stomach. Using this method, overtubes protect the digestive mucosa from injury and limit the risk of aspiration. Additionally, they facilitate access for repeated withdrawal and insertion, as observed in our case. Objects can be extracted through the overtube or retracted into the overtube with both overtube and endoscope removed simultaneously. Though overtubes vary in length (23–135 cm) and diameter (14–21 mm), they must be at least 50 cm in length to ensure both esophagus and stomach are protected [9].

Foreign body removal is the most common indication for overtube use. Potential indications for the use of an overtube during endoscopy include foreign body removal, conduit for endoscopic intubations, reducing looping (in small bowel enteroscopy or difficult colonoscopy), protecting altered anatomy, incorporation with specialized endoscopes (i.e., balloon enteroscopes), access for larger devices (i.e., stent removal, capsule endoscopy delivery, etc.), colonic decompression, and percutaneous endoscopic gastrostomy (PEG) tube placement [9].

The most common complications associated with overtube use are mucosal abrasions and pinching of the mucosal layer between the endoscope and the overtube. Other complications that have been reported include pharyngeal and esophageal perforation, variceal rupture, overtube separation, transient vocal cord paralysis, pneumomediastinum, tracheal compression, and acute pancreatitis [10].

## Conclusions

We report a 27-year-old man who ingested six razor blades and a curtain hook, which were successfully removed using an overtube and a raptor grasping device. Upper endoscopy is the most reliable method for diagnosing and removing foreign bodies. A variety of endoscopic techniques and instruments are available to provide gastroenterologists with therapeutic versatility. While endoscopic techniques are user dependent, an overtube is essential to protect the patients’ airway and facilitates the passage of the endoscope during the removal of multiple ingested objects. Our case also highlights that CT scans may not always detect the actual number of foreign bodies ingested, which could lead to the undermanagement of patients.
